# Crystal structure and Hirshfeld surface analysis of 2-{[(4-iodo­phen­yl)imino]­meth­yl}-4-nitro­phenol

**DOI:** 10.1107/S2056989020008191

**Published:** 2020-06-26

**Authors:** Md. Serajul Haque Faizi, Tenzile Alagöz, Ruby Ahmed, Emine Berrin Cinar, Erbil Agar, Necmi Dege, Ashraf Mashrai

**Affiliations:** a PG Department of Chemistry, Langat Singh College, B. R. A. Bihar University, Muzaffarpur, Bihar 842001, India; b Ondokuz Mayıs University, Faculty of Arts and Sciences, Department of Chemistry, Samsun, Turkey; c Department of Applied Chemistry, ZHCET, Aligarh Muslim University, Aligarh, 202002, UP, India; d Ondokuz Mayıs University, Faculty of Arts and Sciences, Department of Physics, Samsun, Turkey; e Department of Pharmacy, University of Science and Technology, Ibb Branch, Ibb, Yemen

**Keywords:** crystal structure, salicyl­aldehyde derivative, 4-iodo­aniline, 2-hy­droxy-5-nitro­benzaldehyde, hydrogen bonding

## Abstract

The title compound, C_13_H_9_IN_2_O_3_, was synthesized by a condensation reaction between 2-hy­droxy-5-nitro­benzaldehyde and 4-iodo­aniline, and crystallizes in the ortho­rhom­bic space group *Pna*2_1_. The 4-iodo­benzene ring is inclined to the phenol ring by a dihedral angle of 39.1 (2)°. The configuration about the C=N double bond is *E*. The crystal structure features C—H⋯O hydrogen-bonding inter­actions. A Hirshfeld surface analysis of the crystal structure indicates that the most important contributions for the packing arrangement are O⋯H/H⋯O (26.9%) and H⋯H (22.0%) inter­actions.

## Abstract 2

In the title Schiff base compound, C_13_H_9_IN_2_O_3_, the hy­droxy group forms a intra­molecular hydrogen bond to the imine *N* atom and generates an *S*(6) ring motif. The 4-iodo­benzene ring is inclined to the phenol ring by 39.1 (2)°. The configuration about the C=N bonds is *E*. The crystal structure features C—H⋯O hydrogen-bonding inter­actions.

## Chemical context   

Over the past 25 years, extensive research has been directed towards the synthesis and use of Schiff base compounds in organic and inorganic chemistry as they have important medicinal and pharmaceutical applications. These compounds exhibit biological activities, including anti­bacterial, anti­fungal, anti­cancer and herbicidal properties (Desai *et al.*, 2001 ▸; Singh & Dash, 1988 ▸; Karia & Parsania, 1999 ▸). They may also show useful photochromic properties, leading to applications in various fields such as the measurement and control of radiation intensities in imaging systems and optical computers, electronics, optoelectronics and photonics (Iwan *et al.*, 2007 ▸). Schiff bases derived from 2-hy­droxy-5-nitro­benzaldehyde are widely used either as materials or as inter­mediates in explosives, dyestuffs, pesticides and organic synthesis (Yan *et al.*, 2006 ▸). Intra­molecular hydrogen-atom transfer (tautomerism) from the *o*-hy­droxy group to the imine-N atom is of prime importance with respect to the solvato-, thermo- and photochromic properties of *o*-hy­droxy Schiff bases (Filarowski, 2005 ▸; Hadjoudis & Mavridis 2004 ▸). Such proton-exchanging materials can be utilized for the design of various mol­ecular electronic devices (Alarcón *et al.*, 1999 ▸). The present work is a part of an ongoing structural study of Schiff bases and their utilization in the synthesis of quinoxaline derivatives (Faizi *et al.*, 2018 ▸), fluorescence sensors (Faizi *et al.*, 2016 ▸; Mukherjee *et al.*, 2018 ▸; Kumar *et al.*, 2017 ▸; 2018 ▸) and non-linear optical properties (Faizi *et al.*, 2020 ▸). We report herein the synthesis (from 2-hy­droxy-5-nitro­benzaldehyde and 4-iodo­aniline) and crystal structure of the title compound (I), along with the findings of a Hirshfeld surface analysis.
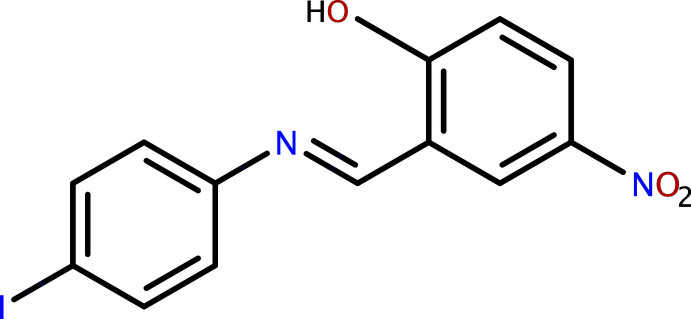


## Structural commentary   

**Figure 1:**
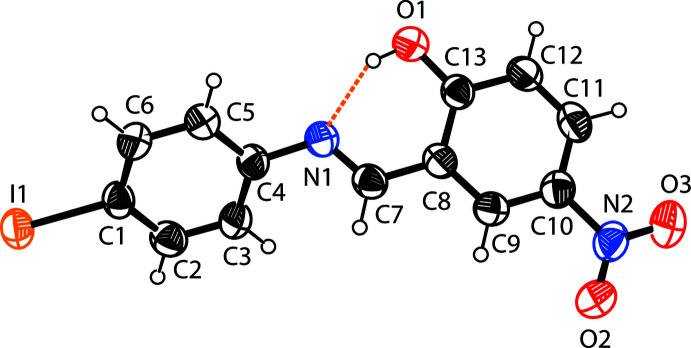
The mol­ecular structure of the title compound, with the atom labelling. Displacement ellipsoids are drawn at the 40% probability level. The intra­molecular N—H⋯O hydrogen bond (see Table1), forming an *S*(6) ring motif, is shown as a dashed line.

The mol­ecular structure of compound (I) is shown in Fig. 1 ▸. An intra­molecular O—H⋯N hydrogen bond is observed (Table 1 ▸ and Fig. 1 ▸). This is a relatively common feature in analogous imine–phenol compounds (see *Database survey* section). The imine group displays a C8—C7—N1—C4 torsion angle of 174.5 (6)°. The 4-iodo­benzene ring (C1–C6) is inclined by a dihedral angle of 39.1 (2)° to the phenol ring (C8–C13), which renders the mol­ecule non-planar. The configuration of the C7=N1 bond of this Schiff base is *E*, and the intra­molecular O1—H1⋯N1 hydrogen bond forms an *S*(6) ring motif (Fig. 1 ▸ and Table 1 ▸). The 4-nitro group is slightly tilted away from co-planarity with the benzene ring to which it is attached [O2—N2—C10—C9 = −7.4 (10)° and O3—N2—C10—C11= −7.4 (10)°]. The C13—O1 distance [1.330 (7) Å] is close to normal for values reported for single C—O bonds in phenols and salicyl­idene­amines (Ozeryanskii *et al.*, 2006 ▸). The N1=C7 bond is short at 1.264 (8) Å, indicative of double-bond character, while the long C7—C8 bond [1.444 (8) Å] implies a single bond. All these data support the existence of the phenol–imine tautomer for (I) in its crystalline state. These features are similar to those observed in related 4-di­methyl­amino-*N*-salicylideneanilines (Filipenko *et al.*, 1983 ▸; Aldoshin *et al.*, 1984 ▸; Wozniak *et al.*, 1995 ▸; Pizzala *et al.*, 2000 ▸). The C—N, C=N and C—C bond lengths are normal and close to the values observed in related structures (Faizi *et al.*, 2017*a* ▸,*b* ▸).

## Supra­molecular features   

**Figure 2:**
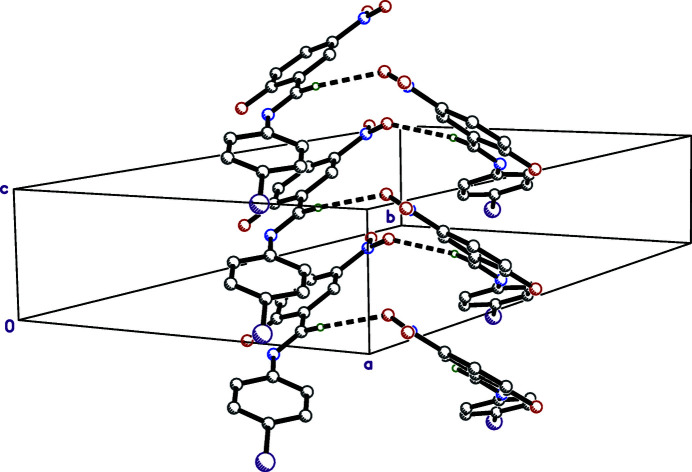
A partial packing plot showing the C—H⋯O hydrogen-bonded (thick dashed lines) helical chains about the crystallographic 2_1_ screw axis parallel to *c*.

**Figure 3:**
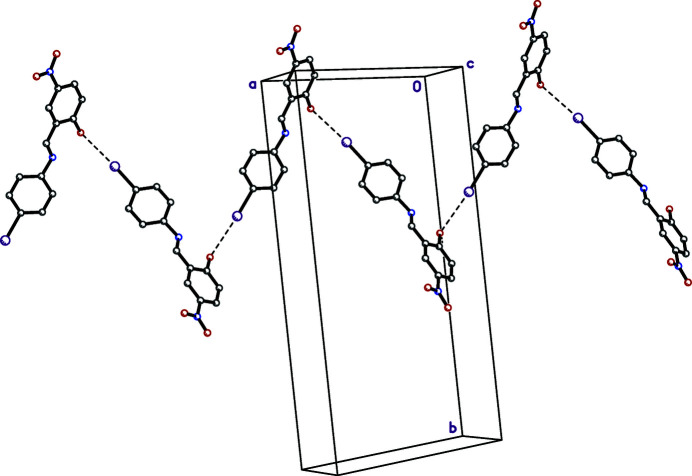
A partial packing plot showing close contacts (dashed lines) between iodine and the phenolic oxygen of glide-related (*x* + 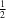, 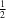 − *y*, −1 + *z*) mol­ecules.

In the crystal packing of (I), the most important inter­molecular inter­actions are weak C7—H7⋯O2^i^ [symmetry code: (i) 1 − *x*, 1 − *y*, −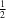 + *z*] hydrogen bonds between screw-related mol­ecules, which form helical chains propagating along the crystallographic screw axis parallel to *c* (Fig. 2 ▸, Table 1 ▸). The shortest inter­molecular contact involving the iodine is 3.351 (5) Å, between glide-related mol­ecules, I1⋯O1^ii^ [symmetry code: (ii) *x* + 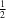, 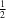 − *y*, −1 + *z*)], which makes a zigzag tape motif (Fig. 3 ▸). There are no other significant inter­molecular inter­actions present in the crystal. The Hirshfeld surface analysis confirms the role of the C—H⋯O inter­actions in the packing arrangement.

## Hirshfeld surface analysis   

**Figure 4:**
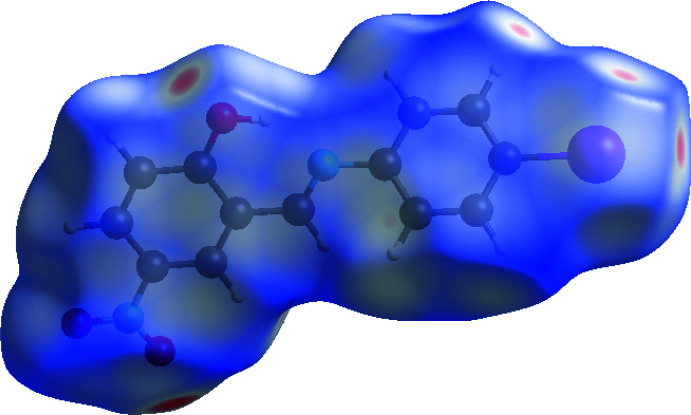
Hirshfeld surface of the title compound plotted over *d*_norm._

**Figure 5:**
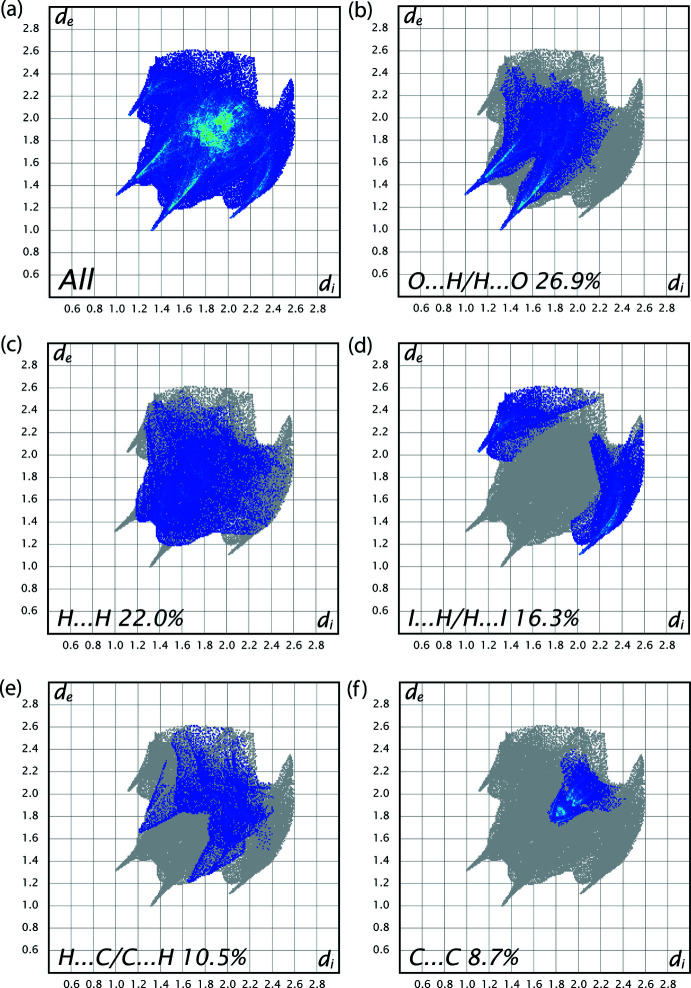
Two-dimensional fingerprint plots of the crystal with the relative contributions of the atom pairs to the Hirshfeld surface along with *d*_norm_ full.

In order to visualize the inter­molecular inter­actions in the crystal packing of (I), a Hirshfeld surface (HS) analysis (Hirshfeld, 1977 ▸; Spackman & Jayatilaka, 2009 ▸) was carried out using *Crystal Explorer 17.5* (Turner *et al.*, 2017 ▸). In the HS plotted over *d*_norm_ (Fig. 4 ▸), white surfaces indicate contacts with distances equal to the sum of van der Waals radii, and the red and blue colours indicate distances shorter (*i.e*., in close contact) or longer than the van der Waals radii sum, respectively (Venkatesan *et al.*, 2016 ▸). The two-dimensional finger print plots are depicted in Fig. 5 ▸. The O⋯H/H⋯O (26.9%) inter­actions form the majority of contacts, with H⋯H (22.0%) inter­actions representing the next highest contribution. The percentage contributions of other inter­actions are: I⋯H/H⋯I (16.3%), C⋯H/H⋯C (10.5%), C⋯C (8.7%), O⋯C/C⋯O (4.7%), N⋯C/C⋯N (3.8%), I⋯C/C⋯I (2.3%), H⋯N/N⋯H (1.4%), I⋯O/O⋯I (2.0%), I⋯N/N⋯I (0.6%), I⋯I (0.5%), O⋯N/N⋯O (0.2%), N⋯N (0.1%) and O⋯O (0.1%).

## Database survey   

A search of the Cambridge Structural Database (CSD, version 5.39; Groom *et al.*, 2016 ▸) gave 26 hits for the (*E*)-2-{[(4-iodo­phen­yl)imino]­meth­yl}-phenol fragment. Of these 26, the most similar to (I), are as follows. In *p*-iodo-*N*-(*p*-cyano­benzyl­idene)aniline (LALMEQ; Ojala *et al.*, 1999 ▸), the OH group is absent and the NO_2_ group is replaced by a cyano group. In (*E*)-5-(di­ethyl­amino)-2-[(4-iodo­phenyl­imino)­meth­yl]phenol (VEFPED; Kaştaş *et al.*, 2012 ▸), the NO_2_ is replaced by an *N*,*N* diethyl group. In *N*-(3,5-di-*tert*-butyl­salicyl­idene)-4-iodo­benzene; (MILFET; Spangenberg *et al.*, 2007 ▸), the NO_2_ group is absent but a pair of ^*t*^Bu groups occupy the 3,5 positions of the salicyl­idene group. In 2-{[(4-iodo­phen­yl)imino]­meth­yl}-6-meth­oxy­phenol (SEDBIP; Carletta, *et al.*, 2017 ▸), the NO_2_ group is absent and a meth­oxy group is *ortho* to the hydroxyl. Lastly, in *N*-(2-cyano­benzyl­idene)-4-iodo­aniline (XOXKIF; Ojala *et al.*, 1999 ▸) the NO_2_ is absent and the OH is replaced by cyano. All these compounds have an *E* configuration about the C=N bond and form the *S*(6) ring motif.

## Synthesis and crystallization   

The title compound was synthesized by condensation of 2-hy­droxy-5-nitro­benzaldehyde (11.0 mg, 0.066 mmol) and 4-iodo­aniline (14.4 mg, 0.066 mmol) in ethanol (15 ml). After the mixture had refluxed for about 15 h, the orange product was washed with ether and dried at room temperature (yield 60%, m.p. 484–486 K). Crystals suitable for X-ray analysis were obtained by slow evaporation of an ethanol solution.

## Refinement   

Crystal data, data collection and structure refinement details are summarized in Table 2 ▸. The OH hydrogen atoms and the C-bound H atoms were included in calculated positions and allowed to ride on the parent atoms: O—H = 0.82 Å, C—H = 0.93–0.96 Å with *U*_iso_(H) = 1.5*U*_eq_(C-meth­yl) and 1.2*U*_eq_(C) for other H atoms.

## Supplementary Material

Crystal structure: contains datablock(s) I. DOI: 10.1107/S2056989020008191/pk2635sup1.cif

Structure factors: contains datablock(s) I. DOI: 10.1107/S2056989020008191/pk2635Isup2.hkl

Supporting information file. DOI: 10.1107/S2056989020008191/pk2635Isup3.cml

CCDC reference: 1922980

Additional supporting information:  crystallographic information; 3D view; checkCIF report

## Figures and Tables

**Table 1:** Hydrogen-bond geometry (Å, °)

*D*—H⋯*A*	*D*—H	H⋯*A*	*D*⋯*A*	*D*—H⋯*A*
O1—H1⋯N1	0.82	1.86	2.591 (6)	148
C7—H7⋯O2^i^	0.93	2.45	3.309 (8)	154

Symmetry code: (i) 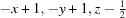.

**Table 2:** Experimental details

Crystal data	
Chemical formula	C_13_H_9_IN_2_O_3_
*M*_r_	368.12
Crystal system, space group	Orthorhombic, *P* *n* *a*2_1_
Temperature (K)	296
*a*, *b*, *c* (Å)	12.8022 (4), 24.4556 (9), 4.1459 (1)
*V* (Å^3^)	1298.02 (7)
*Z*	4
Radiation type	Mo *K*α
μ (mm^−1^)	2.47
Crystal size (mm)	0.42 × 0.34 × 0.21

Data collection	
Diffractometer	Stoe IPDS 2
Absorption correction	Integration (*X-RED32*; Stoe & Cie, 2002 ▸)
*T*_min_, *T*_max_	0.944, 0.981
No. of measured, independent and observed [*I* > 2σ(*I*)] reflections	15403, 2508, 2231
*R*_int_	0.084
(sin θ/λ)_max_ (Å^−1^)	0.617

Refinement	
*R*[*F* ^2^ > 2σ(*F* ^2^)], *wR*(*F* ^2^), *S*	0.037, 0.094, 1.05
No. of reflections	2508
No. of parameters	173
No. of restraints	1
H-atom treatment	H-atom parameters constrained
Δρ_max_, Δρ_min_ (e Å^−3^)	0.81, −0.25
Absolute structure	Flack *x* determined using 814 quotients [(*I* ^+^)−(*I* ^−^)]/[(*I* ^+^)+(*I* ^−^)] (Parsons *et al.*, 2013 ▸)
Absolute structure parameter	0.00 (4)

Computer programs: *X-AREA* and *X-SHAPE* (Stoe & Cie, 2002 ▸), *SHELXT2018/2* (Sheldrick, 2015*a* ▸), *SHELXL2018/3* (Sheldrick, 2015*b* ▸), *ORTEP-3 for Windows* (Farrugia, 2012 ▸) and *XP* in *SHELXTL* (Sheldrick, 2008 ▸).

## supplementary crystallographic information

### Crystal data

**Table:**

C_13_H_9_IN_2_O_3_	*D*_x_ = 1.884 Mg m^−^^3^
*M**_r_* = 368.12	Mo *K*α radiation, λ = 0.71073 Å
Orthorhombic, *P**n**a*2_1_	Cell parameters from 25449 reflections
*a* = 12.8022 (4) Å	θ = 1.7–29.9°
*b* = 24.4556 (9) Å	µ = 2.47 mm^−^^1^
*c* = 4.1459 (1) Å	*T* = 296 K
*V* = 1298.02 (7) Å^3^	Prism, colorless
*Z* = 4	0.42 × 0.34 × 0.21 mm
*F*(000) = 712	

### Data collection

**Table:**

STOE IPDS 2 diffractometer	2508 independent reflections
Radiation source: sealed X-ray tube, 12 x 0.4 mm long-fine focus	2231 reflections with *I* > 2σ(*I*)
Plane graphite monochromator	*R*_int_ = 0.084
Detector resolution: 6.67 pixels mm^-1^	θ_max_ = 26.0°, θ_min_ = 1.8°
rotation method scans	*h* = −15→15
Absorption correction: integration (X-RED32; Stoe & Cie, 2002)	*k* = −30→30
*T*_min_ = 0.944, *T*_max_ = 0.981	*l* = −5→4
15403 measured reflections	

### Refinement

**Table:**

Refinement on *F*^2^	Hydrogen site location: inferred from neighbouring sites
Least-squares matrix: full	H-atom parameters constrained
*R*[*F*^2^ > 2σ(*F*^2^)] = 0.037	*w* = 1/[σ^2^(*F*_o_^2^) + (0.0632*P*)^2^] where *P* = (*F*_o_^2^ + 2*F*_c_^2^)/3
*wR*(*F*^2^) = 0.094	(Δ/σ)_max_ < 0.001
*S* = 1.05	Δρ_max_ = 0.81 e Å^−^^3^
2508 reflections	Δρ_min_ = −0.25 e Å^−^^3^
173 parameters	Absolute structure: Flack *x* determined using 814 quotients [(*I*^+^)-(*I*^-^)]/[(*I*^+^)+(*I*^-^)] (Parsons *et al.*, 2013)
1 restraint	Absolute structure parameter: 0.00 (4)

### Special details

**Table:**

Geometry. All esds (except the esd in the dihedral angle between two l.s. planes) are estimated using the full covariance matrix. The cell esds are taken into account individually in the estimation of esds in distances, angles and torsion angles; correlations between esds in cell parameters are only used when they are defined by crystal symmetry. An approximate (isotropic) treatment of cell esds is used for estimating esds involving l.s. planes.

### Fractional atomic coordinates and isotropic or equivalent isotropic displacement parameters (Å^2^)

**Table:**

	*x*	*y*	*z*	*U*_iso_*/*U*_eq_	
I1	0.50544 (3)	0.16672 (2)	−0.1482 (5)	0.06914 (19)	
O1	0.1141 (3)	0.43513 (17)	0.4285 (13)	0.0721 (13)	
H1	0.148899	0.407604	0.393280	0.108*	
N1	0.2784 (3)	0.37325 (18)	0.4176 (13)	0.0605 (13)	
C8	0.2751 (4)	0.4584 (2)	0.6892 (16)	0.0575 (13)	
C1	0.4310 (5)	0.2352 (2)	0.0459 (14)	0.0578 (12)	
C9	0.3293 (4)	0.4960 (2)	0.875 (2)	0.0614 (12)	
H9	0.397472	0.488588	0.939016	0.074*	
C13	0.1701 (4)	0.4702 (2)	0.6025 (17)	0.0564 (12)	
C11	0.1808 (4)	0.5566 (2)	0.870 (2)	0.0688 (15)	
H11	0.151047	0.589917	0.926484	0.083*	
C6	0.3291 (5)	0.2321 (2)	0.1458 (18)	0.0689 (16)	
H6	0.293084	0.199177	0.130244	0.083*	
C10	0.2824 (4)	0.5444 (2)	0.9636 (16)	0.0618 (15)	
O3	0.3034 (5)	0.6282 (2)	1.207 (2)	0.120 (3)	
N2	0.3421 (4)	0.5833 (2)	1.1596 (16)	0.0751 (16)	
C7	0.3254 (4)	0.4086 (2)	0.5862 (18)	0.0592 (12)	
H7	0.394439	0.402274	0.645609	0.071*	
C3	0.4358 (5)	0.3296 (2)	0.1943 (18)	0.0654 (15)	
H3	0.472183	0.362411	0.211171	0.079*	
C12	0.1245 (4)	0.5192 (3)	0.6944 (17)	0.0655 (15)	
H12	0.055800	0.526809	0.636953	0.079*	
C2	0.4847 (4)	0.2839 (3)	0.067 (2)	0.0679 (16)	
H2	0.553487	0.286056	−0.003196	0.082*	
C4	0.3331 (4)	0.3266 (2)	0.2959 (17)	0.0575 (15)	
C5	0.2798 (4)	0.2777 (2)	0.2694 (15)	0.0660 (18)	
H5	0.210481	0.275532	0.335185	0.079*	
O2	0.4261 (4)	0.5694 (2)	1.2640 (15)	0.0946 (19)	

### Atomic displacement parameters (Å^2^)

**Table:**

	*U*^11^	*U*^22^	*U*^33^	*U*^12^	*U*^13^	*U*^23^
I1	0.0821 (3)	0.0597 (3)	0.0656 (3)	0.01321 (13)	−0.0044 (3)	−0.0083 (2)
O1	0.0590 (19)	0.063 (2)	0.094 (4)	0.0028 (16)	−0.007 (2)	−0.007 (2)
N1	0.061 (2)	0.051 (2)	0.069 (4)	0.0033 (17)	−0.004 (2)	0.002 (2)
C8	0.054 (3)	0.052 (3)	0.066 (4)	0.002 (2)	0.007 (3)	0.004 (2)
C1	0.071 (3)	0.049 (3)	0.054 (3)	0.009 (2)	−0.006 (3)	0.002 (2)
C9	0.054 (2)	0.060 (3)	0.070 (4)	0.0001 (18)	−0.001 (4)	−0.001 (3)
C13	0.056 (3)	0.049 (3)	0.064 (3)	0.001 (2)	0.004 (3)	0.003 (3)
C11	0.067 (3)	0.058 (3)	0.081 (4)	0.007 (2)	0.022 (4)	0.000 (4)
C6	0.070 (3)	0.054 (3)	0.083 (5)	−0.002 (2)	−0.006 (3)	−0.007 (3)
C10	0.064 (3)	0.056 (3)	0.066 (4)	−0.006 (2)	0.010 (2)	−0.001 (2)
O3	0.111 (4)	0.077 (3)	0.171 (8)	0.001 (3)	−0.007 (4)	−0.049 (4)
N2	0.074 (3)	0.066 (3)	0.085 (5)	−0.015 (2)	0.014 (3)	−0.015 (3)
C7	0.057 (3)	0.052 (3)	0.068 (3)	0.004 (2)	−0.001 (3)	0.008 (3)
C3	0.068 (3)	0.049 (3)	0.079 (4)	−0.004 (2)	0.001 (3)	−0.004 (2)
C12	0.054 (3)	0.061 (3)	0.081 (4)	0.006 (2)	0.003 (3)	−0.001 (3)
C2	0.059 (3)	0.069 (4)	0.075 (5)	0.002 (2)	0.005 (3)	0.003 (4)
C4	0.062 (2)	0.046 (2)	0.064 (5)	0.0059 (19)	−0.005 (3)	0.002 (2)
C5	0.060 (3)	0.060 (3)	0.078 (5)	0.003 (2)	−0.003 (3)	−0.005 (3)
O2	0.072 (2)	0.088 (3)	0.124 (6)	−0.012 (2)	−0.008 (3)	−0.028 (3)

### Geometric parameters (Å, º)

**Table:**

I1—C1	2.089 (5)	C11—H11	0.9300
O1—C13	1.330 (7)	C6—C5	1.381 (8)
O1—H1	0.8200	C6—H6	0.9300
N1—C7	1.264 (8)	C10—N2	1.466 (8)
N1—C4	1.430 (7)	O3—N2	1.220 (8)
C8—C9	1.385 (9)	N2—O2	1.208 (8)
C8—C13	1.421 (7)	C7—H7	0.9300
C8—C7	1.444 (8)	C3—C4	1.383 (8)
C1—C6	1.371 (9)	C3—C2	1.385 (10)
C1—C2	1.377 (9)	C3—H3	0.9300
C9—C10	1.377 (7)	C12—H12	0.9300
C9—H9	0.9300	C2—H2	0.9300
C13—C12	1.388 (8)	C4—C5	1.381 (8)
C11—C12	1.373 (10)	C5—H5	0.9300
C11—C10	1.389 (9)		
			
C13—O1—H1	109.5	C11—C10—N2	120.2 (5)
C7—N1—C4	120.5 (5)	O2—N2—O3	123.8 (6)
C9—C8—C13	118.7 (5)	O2—N2—C10	118.8 (5)
C9—C8—C7	120.1 (5)	O3—N2—C10	117.4 (6)
C13—C8—C7	121.2 (5)	N1—C7—C8	121.9 (5)
C6—C1—C2	120.2 (6)	N1—C7—H7	119.1
C6—C1—I1	120.4 (4)	C8—C7—H7	119.1
C2—C1—I1	119.4 (4)	C4—C3—C2	120.2 (5)
C10—C9—C8	120.0 (5)	C4—C3—H3	119.9
C10—C9—H9	120.0	C2—C3—H3	119.9
C8—C9—H9	120.0	C11—C12—C13	120.0 (5)
O1—C13—C12	118.6 (5)	C11—C12—H12	120.0
O1—C13—C8	121.1 (5)	C13—C12—H12	120.0
C12—C13—C8	120.2 (5)	C1—C2—C3	119.8 (6)
C12—C11—C10	119.8 (5)	C1—C2—H2	120.1
C12—C11—H11	120.1	C3—C2—H2	120.1
C10—C11—H11	120.1	C5—C4—C3	119.4 (5)
C1—C6—C5	120.1 (6)	C5—C4—N1	118.5 (5)
C1—C6—H6	119.9	C3—C4—N1	122.1 (5)
C5—C6—H6	119.9	C6—C5—C4	120.2 (5)
C9—C10—C11	121.2 (6)	C6—C5—H5	119.9
C9—C10—N2	118.6 (5)	C4—C5—H5	119.9
			
C13—C8—C9—C10	−1.8 (10)	C4—N1—C7—C8	174.5 (6)
C7—C8—C9—C10	177.8 (7)	C9—C8—C7—N1	179.8 (6)
C9—C8—C13—O1	−179.1 (6)	C13—C8—C7—N1	−0.5 (10)
C7—C8—C13—O1	1.3 (9)	C10—C11—C12—C13	−1.9 (11)
C9—C8—C13—C12	2.0 (9)	O1—C13—C12—C11	−179.1 (7)
C7—C8—C13—C12	−177.7 (6)	C8—C13—C12—C11	−0.1 (10)
C2—C1—C6—C5	−0.2 (10)	C6—C1—C2—C3	0.9 (11)
I1—C1—C6—C5	−179.0 (5)	I1—C1—C2—C3	179.7 (6)
C8—C9—C10—C11	−0.1 (10)	C4—C3—C2—C1	−0.7 (12)
C8—C9—C10—N2	−179.9 (6)	C2—C3—C4—C5	0.0 (11)
C12—C11—C10—C9	2.1 (11)	C2—C3—C4—N1	−177.7 (7)
C12—C11—C10—N2	−178.2 (7)	C7—N1—C4—C5	146.2 (7)
C9—C10—N2—O2	−7.4 (10)	C7—N1—C4—C3	−36.1 (10)
C11—C10—N2—O2	172.8 (7)	C1—C6—C5—C4	−0.6 (10)
C9—C10—N2—O3	172.4 (7)	C3—C4—C5—C6	0.7 (10)
C11—C10—N2—O3	−7.4 (10)	N1—C4—C5—C6	178.5 (6)

### Hydrogen-bond geometry (Å, º)

**Table:**

*D*—H···*A*	*D*—H	H···*A*	*D*···*A*	*D*—H···*A*
O1—H1···N1	0.82	1.86	2.591 (6)	148
C7—H7···O2^i^	0.93	2.45	3.309 (8)	154

Symmetry code: (i) −*x*+1, −*y*+1, *z*−1/2.
